# Exosomes derived from human adipose mensenchymal stem cells accelerates cutaneous wound healing via optimizing the characteristics of fibroblasts

**DOI:** 10.1038/srep32993

**Published:** 2016-09-12

**Authors:** Li Hu, Juan Wang, Xin Zhou, Zehuan Xiong, Jiajia Zhao, Ran Yu, Fang Huang, Handong Zhang, Lili Chen

**Affiliations:** 1 Department of Stomatology, Union Hospital, Tongji Medical College, Huazhong University of Science and Technology, Wuhan, Hubei 430022, China

## Abstract

Prolonged healing and scar formation are two major challenges in the treatment of soft tissue trauma. Adipose mesenchymal stem cells (ASCs) play an important role in tissue regeneration, and recent studies have suggested that exosomes secreted by stem cells may contribute to paracrine signaling. In this study, we investigated the roles of ASCs-derived exosomes (ASCs-Exos) in cutaneous wound healing. We found that ASCs-Exos could be taken up and internalized by fibroblasts to stimulate cell migration, proliferation and collagen synthesis in a dose-dependent manner, with increased genes expression of N-cadherin, cyclin-1, PCNA and collagen I, III. *In vivo* tracing experiments demonstrated that ASCs-Exos can be recruited to soft tissue wound area in a mouse skin incision model and significantly accelerated cutaneous wound healing. Histological analysis showed increased collagen I and III production by systemic administration of exosomes in the early stage of wound healing, while in the late stage, exosomes might inhibit collagen expression to reduce scar formation. Collectively, our findings indicate that ASCs-Exos can facilitate cutaneous wound healing via optimizing the characteristics of fibroblasts. Our results provide a new perspective and therapeutic strategy for the use of ASCs-Exos in soft tissue repair.

While soft tissue trauma is a common occurrence, many chronic wounds, such as diabetic ulcers^1^, are difficult to heal^2^. Prolonged healing soft tissue wound are more likely to form scars, which means less favorable healing quality^3^,^4^. This not only causes functional disability, but also can affect the mental health of patients. Therefore, shortening healing time and reducing the scar formation in soft tissue trauma recovery represent urgent clinical needs. Current conventional methods to accelerate the healing and reduce scar formation include skin grafting^5^,^6^, laser therapy^7^ and the local application of some growth factors^8^ or gene therapy^9^. However these methods may lead to atrophic scar, pigment abnormalities, skin necrosis and other undesirable consequences^10^. Additionally, local injected factors can easily be degraded by body fluid, and their dosage and concentration are often highly variable at wound site^11^. Hence, it is necessary to find a new stable, efficient and safe method to promote soft tissue wound healing.

Fat tissue is widely distributed in human body, and plays a vital role in supporting and protecting adjacent soft tissue in physiological state^12^. At the same time, as an active endocrine organ, fat tissue is also crucial element of metabolism, growth and development, inflammation resistance, among other biological processes^13^,^14^. In recent years, fat tissue has been extensively studied for its role in wound repair. Autologous adipose graft was applied for complicated wound repair^15^, and also often used to regenerate soft tissues in plastic surgery. Its efficacy andsafety are widely accepted^16^,^17^, but there is lack of direct evidence for its mechanism.

Exosomesare a kind of membrane lipid vesicles with 30–100 nm in diameter, and they were previously thought to be metabolic products of cells^18^,^19^. According to previous studies, exosomes represent an essential medium for intercellular communication as a variety of miRNAs and proteins are sorted in exosomes^20^,^21^,^22^. Studies have demonstrated that exosomes derived stem cell may aid in tissue repair due to their advantages of high stability, non-immune rejection, homing effect, easy control of dosage and concentration^23^,^24^,^25^. Exosomes secreted by umbilical cord mesenchymal stem cells can effectively repair the damage of myocardial ischemia reperfusion injury^26^,^27^, liver fibrosis^28^ and acute kidney injury^29^. In the pathogenesis and progression of breast cancer, exosomes secreted by adipose mesenchymal stem cells (ASCs-Exos) can affect cancer cell migration through Wnt signaling pathway. ASCs-Exos also contain a high level of enkephalinase, which is helpful for Alzheimer treatment. In our previous study, we found that conditioned medium of cultured adipose stem cells (ASC-CM) could promote migration, proliferation and collagen synthesis of fibroblasts^30^, and we extracted a number of exosomes from the conditioned medium. Herein, we hypothesized that exosomes secreted by adipose derived stem cells have a positive role in promotion of skin or mucosal soft tissue wound repair. To address this hypothesis, we compared the healing rate of wound with fat layer and without fat layer, and examined the expression of exosomes in these two sites of wound. *In vitro and in vivo* tracking of exosome were also explored. We found that exosomes derived from ASCs can result in changes to cell proliferation, migration and collagen synthesis, which can benefit wound healing. This study confirmed that ASCs-Exoscan shorten the healing time and reduce scar formation of mouse skin incision wound. These data present strong *in vitro* and *in vitro* evidence that ASCs-Exos have promising potential for clinical application in soft tissue wound healing.

## Results

### Subcutaneous fat tissue was benefit for skin wound healing

**Figure 1:**
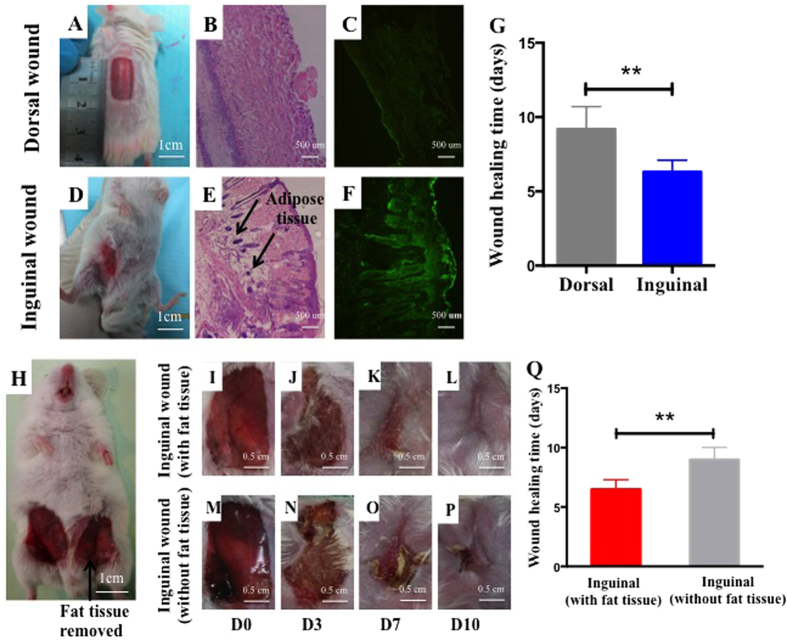
Subcutaneous fat tissue was beneficial for skin wound healing. (**A**) The same size of wounds (1.5 × 1 cm) were created respectively on the back and groin area (**D**) of mice. H&E staining showed that abundant fat tissue existed under inguinal wound (**B**), while no obvious fat tissue existed under dorsal wound (**E**). Immunofluorescence staining indicated wide distribution of CD63 in inguinal wound area (**C,F**). (**G**) Dorsal wound took longer time to completely heal than inguinal wound (**P < **0.01**). The samesize of wounds (1.5 × 1 cm) were also created respectively on the two sides of groin areaof each mouse, and the fat layer of one side wound was removed (**H**). Pictures for wound area of two sides wound showed that wound with fat layer healed faster than inguinal wound without fat layer (**I–P,Q**).

To investigate the influence of subcutaneous fat tissue on skin wound healing, we performed a preliminary study to comparethe healing rate of skin wounds with or without a fat layer. Briefly, we created the same size of inguinal wound (with fat layer) and dorsal wound (without fat layer) on mice (Fig. 1A,B,D,E). Our results demonstrated that CD63, a specific marker of exosomes, was expressed at significantly higher levels in the inguinal wound area (Fig. 1C,F). Moreover, the dorsal wound took a longer time to completely heal than inguinal wound (p < 0.01), (Fig. 1G). We also compared the wound healing of a inguinal wound with fat layer and a inguinal wound without fat layer (fat tissue was removed form the wound) on same mouse (Fig. 1H), and found that inguinal wound with fat layer healed better than inguinal wound without fat layer, as the former wound performing less scar and shorter healing time (Fig. 1I–P and Fig. 1Q). To exclude the possible effects of the intrinsic differences between the inguinal and dorsal fibroblasts on wound healing, we cultured mice inguinal and dorsal fibroblasts separately *in vitro*, and compared their proliferation and migration abilities. The results presented no difference between these two fibroblasts (Supplementary Fig.1). These results suggest that subcutaneous fat tissue was beneficial for skin wound healing, and this phenomenon might be closely relevant with the production of exosomes in fat layer.

### Characterization of human ASCs-Exos

**Figure 2:**
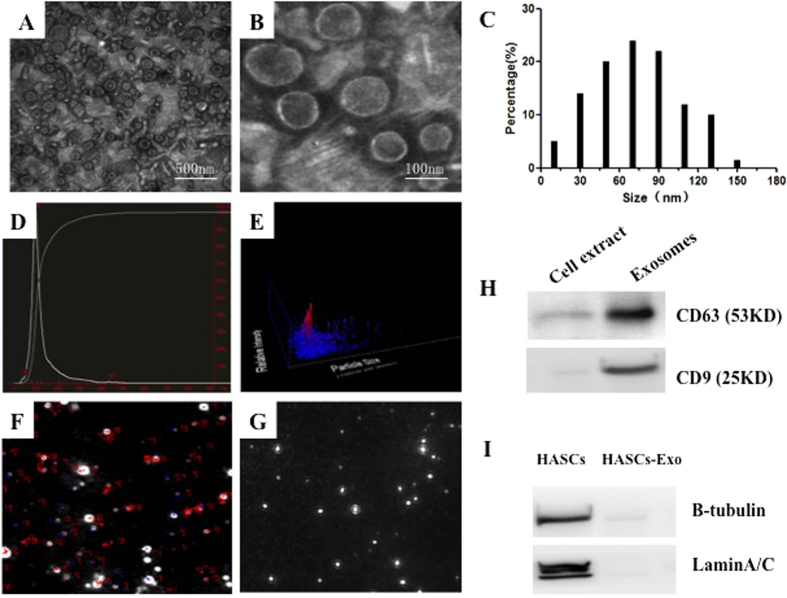
Characterization of human ASCs-Exos. Microscope images of human ASCs-Exos morphology(**A,B**). These particles size distribution displayed about 85% range from 30 to 100 nm (**C**). Characterizations of exosomes were measured by NanoSight analysis (**D–G**). Size distribution (**D**), concentration analysis (**E**), vesicles’ trajectory of Brownian motion (**F**), and Dynamic tracking video capture (**G**). Detection of CD63 and CD9 expression in exosomes by western blotting (**H**). The purity of ASCs was detected by western blotting analysis of Tubulin (cytosolic marker) andLamin A/C (nuclear marker)(**I**).

We isolated exosomes from the supernatants of ASCs. The cup-shaped morphology of exosomes was observed by electron microscopic analysis,and their diameters almost ranged from 30 to 100 nm (Fig. 2A–C). The particle size distribution, concentration and dynamic tracking were measured by using NanoSight analysis (Fig. 2D–G). Western blot showed some exosomal markers, such as CD9 and CD63 (Fig. 2H), were expressed in exosomes. To detect the purity of the exosome isolated from ASCs, we measured the expression of Tubulin (cytosolic marker) and Lamin A/C(nuclear marker) in the ASCs and exosomes by western blot. A barely detectable expression of Tubulin and Lamin A/C were presented in exosomes, which means no contamination of cellular components in isolated exosomes (Fig. 2I). The results indicated that exosomes from human ASC were successfully isolated and consistent with the defined exosomes. Fibroblasts also can secrete exosomes, and the concentrations of exosomes secreted by equal amount of mice inguinal ASCs, inguinal fibroblasts and dorsal fibroblasts are nearly the same (Supplementary Fig.2), but the components of exosomes secreted by these cells are theoretically different, which needs further study.

### Internalization of exosomes by fibroblasts

**Figure 3:**
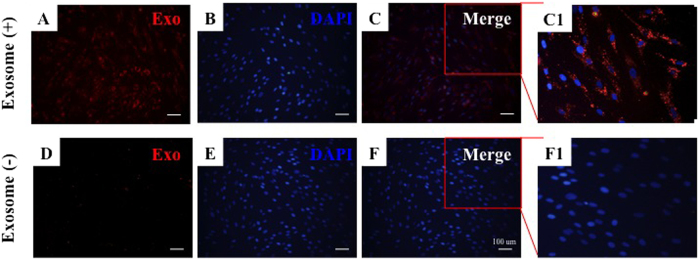
Cellular internalization of ASCs-Exos by fibroblasts. Fibroblasts were incubated with CM-Dil dye labeled exosomes for 24 h (**A–C**), and cells were also incubated with dye without exosomes as a negative control to examine carryover of CM-Dildye (**D–F**). Numerous dye labeled exosomes were observed inside the fibroblasts (**C1**), while there was no dye present in the control group (**F1**).

Studies have reported that exosomes can enter into target cells, thereby regulating their biological behavior. As such, we investigated whether exosomes from human ASCs can enter into fibroblasts. In our *in vitro* tracking experiment, the labeled exosomes were incubated with fibroblasts for 24 h, and the cellular uptake of ASCs-Exos was evaluated via fluorescence microscopy (Fig. 3A–C1). This analysis demonstrated that exosomes can enter into the cytoplasm of fibroblasts, mainly localizing to the perinuclear region, implying that ASCs-Exos can be internalized by fibroblasts.

### ASCs-Exos promoted fibroblasts migration, proliferation, collagen synthesis *in vitro*

**Figure 4:**
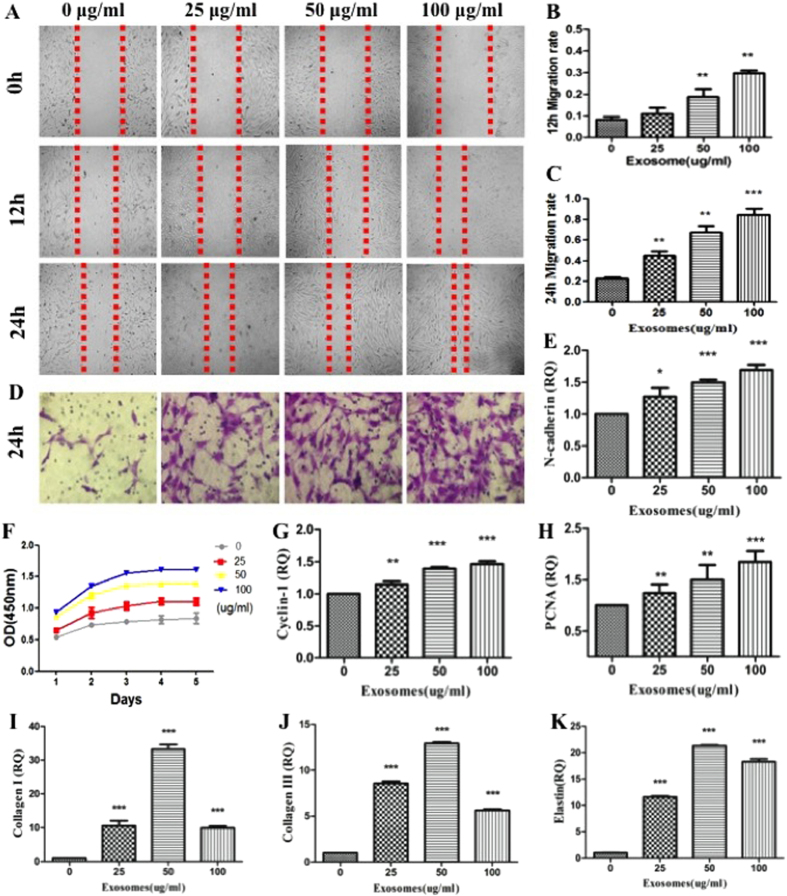
The changes of characteristics of human dermal fibroblasts with stimulation of exosomes. The images (**A**) and migration rates of human fibroblasts following co-culture with 0, 25, 50, or 100 μg/mL ASCs-Exos for 12 h (**B**) or 24 h (**C**). Transwelltest of fibroblasts with stimulation of different concentrations of exsomes (**D**). Growth curves of fibroblasts co-cultured with 0, 25, 50, or 100 μg/mL ASC-Exos (**F**). The N-calcium (**E**) Cyclin-1 (**G**) and PCNA (**H**) mRNA expression of human fibroblasts. The Col I (**I**), III (**J**) and elastin (**K**) mRNA expression of human fibroblasts treated with ASCs-Exos.*P < 0.05, **P < 0.01, ***P < 0.001.

Our previous studies have found that human adipose stem cells conditioned medium (ASCs-CM) can significantly promote human skin dermis fibroblasts (FBs) migration, proliferation and collagen synthesis. We hypothesized that this effect may be partly due to the participation of exosomes. Scratch closure test results demonstrated that the migration of fibroblasts increased at 12 h and 24 h in the presence of exosomes as compared to control group p < 0.001) (Fig. 4A–C). Similarly, a Transwell migration assay detected that exosomes could promote fibroblasts migration in a dose-dependent manner (Fig. 4D). CCK-8 analysis of cell proliferation showed that exosomes also promoted fibroblasts proliferation with the increase of concentration of exosomes at Day 1, 2, 3, 4, 5 (Fig. 4F). In addition, mRNA expression of N-cadherin (Fig. 4E), Cyclin-1 (Fig. 4G) and PCNA (p < 0.001, Fig. 4H) suggested that increased exosome concentration significantly induced expression of genes related to cell migration and proliferation, respectively.

Further *in vitro* studies were performed to determine that whether exosomes can affect collagen synthesis ability of dermal fibroblasts. After stimulating fibroblasts with ASCs-Exos, Collagen I (Fig. 4I), III (Fig. 4J) gene expression and elastin protein production (Fig. 4K) were significantly increased dose-dependently (p < 0.05), and the optimal concentration of exosomesis 50 ug/ml.

### *In vivo* tracking of intravenous injected exosomes

**Figure 5:**
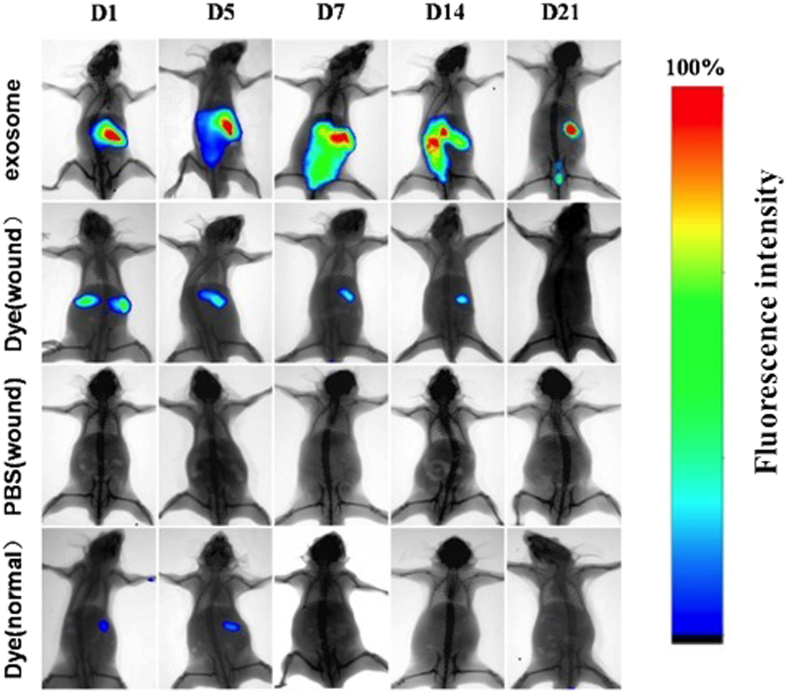
Tracking experiments *in vivo*. Assessment of bioluminescence imaging signals evaluate whether ASCs-Exos can migrate to the wound site. Representative bioluminescence imaging of animals injected with fluorescent labeled exosomes. Dye or PBS after sufferring a back wound (2 × 1.5 cm) were collected at indicated time points of Day 1, 5, 7, 14, 21. To observe the metabolism of dyes in normal mice, dye was injected into the tail vein of the control group.

To evaluate the contributions of exosomes in wound healing, we monitored the migration and effect of exosomes after they were injected into mice sufferring a back wound. As a control, dye without exosomes was also injected into normal and wounded mice to observe their metabolic processes. Bioluminescence imaging showed (Fig. 5) that fluorescence gathered in the area of the wound in 7 days (D7) and could still be detected until day 21 (D21) when the fluorescence became weak and gradually disappeared. No fluorescence signal wasdetected in control mice where only Dye was injected.

### ASCs-Exos promoted cutanenous wound healing *in vivo*

**Figure 6:**
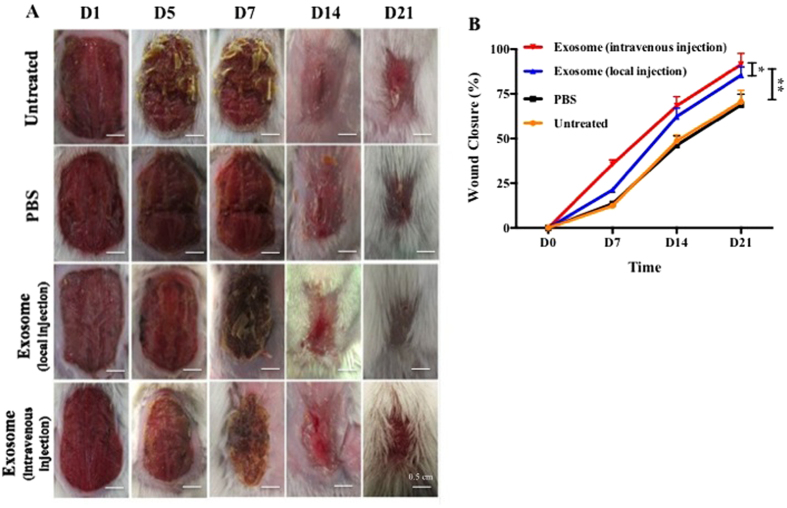
ASCs-Exos promoted wound healing *in vivo*. Representative wound closure imaging of animals injected locally or intravenously with exosomesor PBS after sufferring a back wound (2 × 1.5 cm) were collected at indicated time points of Day 1, 5, 7, 14, 21 (**A**). Untreated and PBS injection severed as control. Quantitative analysis of wound closure at different time points (**B**). *P < 0.05, **P < 0.01.

As shown in Fig. 6A,B, wound closure of exosomes treatment mice was accelerated, illustrated by smaller wound areas measured at day1, 5, 7, 14, and 21 post-wounding when compared with control groups(untreated and PBS-treated). Most notably,intravenous exosomes injection resulted in a 50% closure by day 7 post-wound,was ~75% closed by day 14 and ~90%closed by day 21. Wound healing occurred significantly faster when injected with exosomes than local injection group(p < 0.05).

### ASCs-Exos exosomes promoted collagen expression during wound healing

**Figure 7:**
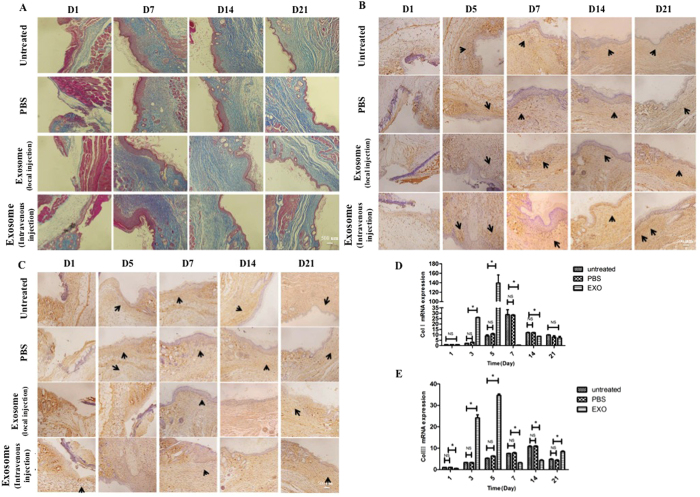
ASCs-Exos promoted collagen expression and secretion during wound healing *in vivo*. Evaluation of collagen synthesis secretion of wounds following treatment with PBS, injected locally or intravenously with exosomesat Day 1, 7, 14, 21 post-wounding, untreated animals served as control (**A**). Immunohistochemical and RT-PCR analysis of collagen synthesis of fibroblasts. The results of immunohistochemical analysis of collagen I (**B**) and collagen III (**C**) were same as above (arrows indicate Col I or Col III positive), withcollagen I (**D**) and collagen III (**E**)were obviously upregulated in the early stage. *P ≤ 0.05; NS: no significant difference.

To evaluate scarless wound healing, we detected collagen deposition by Masson’s staining, showing that the collagen production by Day 7 and the collagen maturity in the late stage of day 14 and 21 in the intravenous injection groups and local injection groups were higher than PBS groupsand untreated groups, respectively (Fig. 7A).

We also performed Immunohistochemical observation and qRT-PCR analysis to assess collagen I (Fig. 7B,D) and III (Fig. 7C,E) expressions in the wound area. Our results showed that these two types of collagen were expressed higher in the groups that received intravenous injection, and reached a peak value at day 5. These results suggested that exosomes can promote the expression of collagen I and III in the early stage of wound healing. While in the late stage, exosomes might inhibit collagen expression.

## Discussion

Fat tissue, which contains a lot of mesenchymal stem cells, is an active endocrine organ, and is thought to play an important role in the repair of soft tissue trauma^31^,^32^. However, there is no direct evidence for this effect, and the underlying mechanism remains enigmatic. Recent studies have demonstrated that exosomes are very important for paracrine activity of stem cells^33^. In this study, the effects of ASCs-Exos on fibroblasts activity and skin wound repair were explored.

The results of our study demonstrate that inguinal wound (with fat layer) healed faster, and had more exosomes distribution in the surrounding of the wound regionthan those with dorsal wound (without fat layer), indicating that both that the presence of a fat layer was beneficial for soft tissue wound healing and that exosomes of adipose tissue may promote this effect.

Exosomes are small membrane vesicles that form by the inward budding of cellular compartments that fuses with the plasma membrane^34^,^35^. Fibroblasts are the main effector cells in the wound healing of soft tissue^36^. Their migration, proliferation and collagen synthesis are important for the quality of wound healing^37^. We found that human ASCs-secreted exosomes can be internalized by fibroblasts anddose-dependently optimized their characteristics, such as the migration and proliferation abilities, collagen synthesis and elastic secretion capacity. The role of exosomes, derived from stem cell, in promoting tissue repair hasbeen reported by numerous sources. Exosomes secreted by umbilical cord mesenchymal stem cells have positive effects on migration,proliferation and tube formation of endothelial cells^38^, while iPS-Exos(human induced pluripotent stem cells) can promote collagen synthesis^39^. It is believed that exosomes are able to exert this affect due to their rich composition of of RNAs and proteins, which are related to the functions of fibroblasts.

Our *in vitro* tracking results showed that exosomes could enter into the cytoplasm of fibroblasts. Similar observations have previously been reported in other cell types. Ramesh *et al*., co-cultured dye labeled exosomes of tumor cells with different cell lines, and confirmed the physical entry of exosomes into the cell cytoplasm^40^. Lipid raft-mediated endocytosis is responsible for exosomes uptake through ERK1/2-Heat Shock Protein 27 Signaling^41^. Reports suggest that miRNAs and proteins derived from exosomes can mediate signal transduction in target cells following endocytosis, or membrane fusion^42^. Our *in vitro* tracking results confirmed that ASCs-Exos released their active substances after they entered into cells to promote migration, proliferation and collagen secretion of fibroblasts.

Local injection of exosomes has also been reported to promote regeneration of damaged tissue, but there is no report regarding the effects of intravenous injection of exosomes. Our *in vivo* tracing experiment found that exosomes can be recruited to wound area via tail vein blood circulation, assembling around the wound on day 7 post-injection, aiding in the healing process. This phenomenon might be similiar to the homing function of stem cells. The cytomembrane of stem cells invaginates into the cytoplasm, and coats miRNAs and proteins to form multivesicular endosome, which combines with cytomembrane to release exosomes to surround environment^43^. Due to surface receptors and adhesion molecules of stem cells^44^,^45^, exosomes may present similar homing abilities as their parent cells, and can be recruited to the wound region. It has been postulated that exosomes can avoid recognition/detection by the immune system, and maintain the integrity of cell membrane to avoid degradation. However, the specific mechanisms through which exosomes contributes to wound healing requires further investigation.

We applied exosomes to mouse skin wound through local injection and intravenous injection, and found that the exosome-treated mice healed faster than the control mice, indicating that exosomes derived from human adipose mensenchymal stem cells can accelerate cutaneous wound healing. Interestingly, intravenous injection was superior at wound healing as compared to local injectionand we speculated loss of exosomes during local injection may contribute to this difference. Moreover, when injecting exosomes directly into the wound, inevitably the wound can be further disturbed, thus disrupting the wound healing process. After entering into blood, exosomes could be recruited to damage area through receptors or adhesion molecules of membrane surface, targeted fibroblasts to promote wound healing by. *In vivo* studies showed that collagen I and III distributions were promoted by exosomes in the early stage of wound healing, a result that was confirmed by with increased expressions of collagen I and III. These results suggest that exosomes promote the early stages of wound healing by shortening healing time, while in the late stages, they might inhibit collagen synthesis to reduce scar formation. This tendency follows the histological changes observed during natural healing of soft tissue wounds, that is, in the early phase of healing, collagen deposition is more important, while in the late phase of healing, matrix reconstruction is more critical.

In summary, exosomes secreted by human adipose stem cells are easily obtained and can be effectively used in research and clinical treatment. Exosomes can optimize the characteristics of fibroblasts, such as promoting the migration, proliferation and collagen synthesis of fibroblasts, thereby accelerating wound healing of soft tissue. Our findings suggest that ASCs-Exos may represent a novel therapeutic tool in soft tissue wound healing.

## Methods and Materials

### Isolation and identification of exosomes derived from human adipose stem cells(HASCs)

HASCs were isolated as previously described^46^. Human subcutaneous fat tissue was obtained from healthy mother aged from 18 to 30 years old with informed consent approved by the Committee of Wuhan Union Hospital and the following protocols including all relevant details were performed in accordance with the Ethics Committee of Tongji Medical College, Huazhong University of Science and Technology (IORG No: IORG0003571, and the ethics statement is in the supplementary information). HASCs were cultured in serum-free medium for 24 hours to collect conditioned medium, and cell debris were removed by centrifuging at 3,000 g for 15 min, and then passed through a 0.22 um filter (Millipore). Supernatants were concentrated using 100 KDa molecular weighAmicon^®^ Ultra-15 Centrifugal Filter Devicest (Millipore) and then incubated with ExoQuick-TC exosome precipitation solution (System Biosciences) overnight. Exosome pellets were resuspended in PBS, and the purified exosomes were passed through a 0.22 um filter. After diluted with PBS, 1 ul exosomes were used to quantitate their concentration by BCA protein assay kit, as suggested by the manufacturer(Beyotime Instituteof Biotechnology). The collected exosomes morphologies were observed by 100 kv transmission electron microscopy (HITACHIH-7000FA, Japan). The size, concentration and particle size distribution of exosomes were identified by NanoSight LM10 (Nanosight) and Nanoparticle Tracking Analysis software version 2.2 (NanoSight). Antibodies against CD9 (Abcam) and CD63 (Abcam) proteins were used to analyse their expression by western blotting.

### Scratch closure test

Primary human dermal fibroblasts were isolated using previously described protocols^47^. 3 × 10^5^ Skin fibroblasts were seeded into 6-well platesand scratched by a sterile 100 ul pipette tip. Fresh serum-free culture medium containing ASCs-Exos (0, 25, 50, 100 ug/ml) were added. We took images of the scratched area at 0, 12, and 24 h, andmeasured widths of the scratched by the Image-Pro Plus 6.0 software.

### *In vitro* Migration Assay

The migration of fibroblasts exposed to exosomes was also determined by Transwell assays using transwell chambers with 8 um pore filters (USA), according to the manufacturer’s recommendation. Approximately 1 × 10^5^ fibroblasts suspended were seeded into the upper compartment and ASCs-Exos (0, 25, 50, 100 ug/ml) were added into the lower compartment. Cells were allowed to migrate for 24 hours, after which the upper chamber was washed three times and wiped toremove nonmigrated cells. Migrated cells were stained with 0.4% crystal violet, and cell counts were performed via microscopy.

### Cell proliferation assay

We used Counting Kit-8 (CCK-8) (Dojindo) to determine proliferation of skin fibroblasts when they were co-cultured with ASCs-Exos, according tothe previously described protocols^47^.

### Collagen synthesis of fibroblasts with exosomes stimulation

Fibroblasts were serum starved for 24 h, and different concentrations of ASCs-Exos (0, 25, 50, 100 ug/ml) with fresh serum-free culture medium were changed for additional 48 hours culture, and cells were collected and collagen expression level was examined by qRT-PCR. The primer sequences were listed in Table 1.

### *In vitro* Tracking

Human ASCs-Exos were fluorescently labeled with CM-Dil dye(CM-Dil;Molecular Probes). The labeled exosomes were passed through 100-kDa filter (Microcon YM-100, Millipore) and resuspended in PBS for 3 times to remove excess dye. Mixtures were co-cultured with fibroblasts in FBS-free medium overnight at 37 °C. Following fixed with 4% paraformaldehyde and 4′, 6-diamidino-2-phenylindole (DAPI, sigma), the cells were observed under a fluorescence microscope.

### Mice skin wound model and treatment

Adult male Balb/c mice (6–8 weeks) were purchased from the Animal Centre of disease control and prevention. All animal procedures including all relevant details were performed in accordance with the Animal Care Committee of Tongji Medical College, Huazhong University of Science andTechnology and approved by Ethics Committee of Tongji Medical College, Huazhong University of Science andTechnology (IORG No: IORG0003571)

Mice model of skin wound was established as previously described. After anesthetizing and shaving the mice, a dorsal wound and an inguinal wound with same size(1 × 1.5 cm) were created. Half the mice were sacrificed at day 7 post-surgery, and the tissues of wound area were cut for H&E staining. CD63 staining of the wound area was analyzed by immunofluorescence. The remaining micewere observed to compare the healing rate of dorsal wound and inguinal wound.

We created a 2 × 1.5 cm full-thickness wound on the back of mice to confirm the effects of ASCs-Exos on cutaneous wound healing. Mice were randomly divided into four groups: untreated group, 200 μl PBS subcutaneous injection group, 200 μg exosome in 200 μl PBS subcutaneous injection group, 200 μg exosome in 200 μl PBS intravenous injection group. The animals were housed individually, and were imaged prior to surgery and regularly everyday post-surgery. Mice were killed at day 1, 3, 5, 7, 14, and 21 following treatment, and wound areas were measured and calculated using image analysis software. Half of the wound skin tissues were collected for qRT-PCR analysis. The rest of the half sampleswere used for immunohistochemistry staining.

### *In vivo* tracking

ASCs-Exos were labeled with DIR (DIR;Molecular Probes) according to the manufacturer’s protocol. Labeled exosomes, dye mixture (not contain exosomes) and PBS were tail intravenously injected into Balb/c mice with a 1.5 × 2 cm dorsal wound, and intravenous injection of pure dye mixture into normal mice were used to as negative control. Mice were anesthetized for observation under bioluminescence system at Day 1, 3, 7, 14 and 21, and fluorescence images for exosomes distribution were acquired with 740 nm excitation and 790 nm emission filters.

### Real-Time PCR

Total RNA was extracted from fibroblasts with the RNeasy Mini Kit (Qiagen), and then reversed-transcribed into complementary DNA (cDNA), and the appropriate primers for PCR were designed as shown in Table1.

### Immunohistochemistry

For immunohistochemical staining of collagen synthesis of fibroblasts, formalin-fixed and paraffin embedded skin tissues were deparaffinized to prevent non-specific protein binding. Samples were then incubated with diluted primary antibodies overnight at 4 °C. After the wash steps, the sections were incubated with secondary antibodies for 2 h. Antibody binding of tissue sections were visualized by incubating with DAB substrate, and counterstained with hematoxylin before the slides were mounted. The collagen expressions were evaluated by high-power light microscopy examination.

### Statistical analyses

The data were assessed using Student’s t-tests (t-tests), one-way or two-wayanalysis of variance (ANOVA) comparing the differences between groups by GraphPad Prism 5 software. P values < 0.05 were considered statistically significant. *P < 0.05, **P < 0.01, ***P < 0.0001. The values are shown in the figures.

## Additional Information

**How to cite this article**: Hu, L. *et al*. Exosomes derived from human adipose mensenchymal stem cells accelerates cutaneous wound healing via optimizing the characteristics of fibroblasts. *Sci. Rep.* **6**, 32993; doi: 10.1038/srep32993 (2016).

## Supplementary Material

Supplementary Information

## Figures and Tables

**Table 1:** Primer Sequences and Products of Reverse Transcription–Polymerase Chain Reaction.

Gene	Forward primer (5′ to 3′)	Reverse primer (5′ to 3′)
Col1a1 (mus)	AAGAAGCACGTCTGGTTTGGAG	GGTCCATGTAGGCTACGCTGTT
Col3a1 (mus)	GTGGCAATGTAAAGAAGTCTCTGAAG	GGGTGCGATATCTATGATGGGTAG
GAPDH (mus)	AGGAGCGAGACCCCACTAACA	AGGGGGGCTAAGCAGTTGGT
Col1a1 (homo)	CAAGACGAAGACATCCCACCAATC	ACAGATCACGTCATCGCACAACA
Col3a1 (homo)	TCGCTCTGCTTCATCCCACTAT	CTTCCAGACATCTCTATCCGCAT
ELASTIN (homo)	GGGTTGTGTCACCAGAAGCA	CAACCCCGTAAGTAGGAATGC
PCNA (homo)	AGCCATATTGGAGATGCTGTTG	CTGAGTGTCACCGTTGAAGAAGAGAG
N-Cadherin(homo)	AAGAGGCAGAGACTTGCGAAAC	TGGAGTCACACTGGCAAACCTT
Cyclin-D1(homo)	GCATCTACACCGACAACTCCATC	CGCGTGTTTGCGGATGATCT
GAPDH (homo)	GGCACAGTCAAGGCTGAGAATG	ATGGTGGTGAAGACGCCAGTA
