# Author Correction: Exosomes derived from human adipose mensenchymal stem cells accelerates cutaneous wound healing via optimizing the characteristics of fibroblasts

**DOI:** 10.1038/s41598-020-63068-7

**Published:** 2020-04-16

**Authors:** Li Hu, Juan Wang, Xin Zhou, Zehuan Xiong, Jiajia Zhao, Ran Yu, Fang Huang, Handong Zhang, Lili Chen

**Affiliations:** Department of Stomatology, Union Hospital, Tongji Medical College, Huazhong University of Science and Technology, Wuhan, Hubei 430022 China

Correction to: *Scientific Reports* 10.1038/srep32993, published online 12 September 2016

This Article contains errors in Figure 4D and Figure 7.

In Figure 4D, the image for 25 µg/ml exosomes panel is incorrect.

In Figure 7, the images for untreated D14, local injection D7, 14, 21, and intravenous injection D14, D21 of Figure 7B are incorrect; The images for untreated D5 and intravenous injection D1 of Figure 7C are incorrect.

The corrected Figures 4D and 7 appear below as Figures 1 and 2.

**Figure 1:**
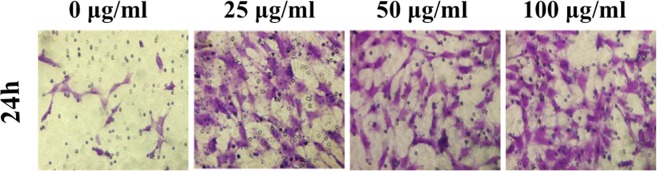
Transwell test of fibroblasts with stimulation of different concentration of exosomes for 24 hours.

**Figure 2:**
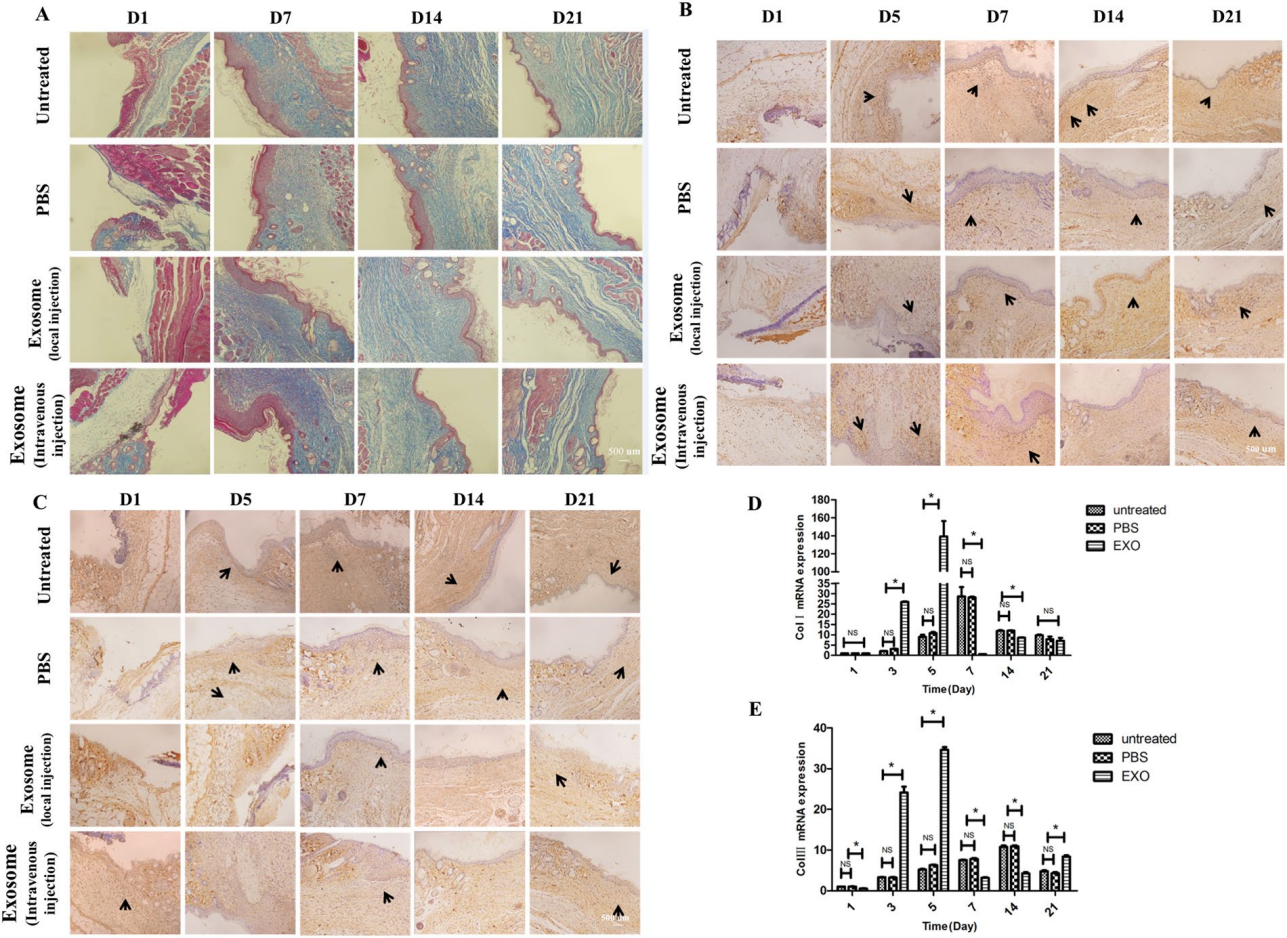
ASCs-Exos promoted collagen expression and secretion during wound healing *in vivo*. Evaluation of collagen synthesis secretion of wounds following treatment with PBS, injected locally or intravenously with exosomes at Day 1, 7, 14, 21 post-wounding, untreated animals served as control (**A**). Immunohistochemical and RT-PCR analysis of collagen synthesis of fibroblasts. The results of immunohistochemical analysis of collagen I (**B**) and collagen III (**C**) were same as above (arrows indicate Col I or Col III positive), with collagen I (**D**) and collagen III (**E**) were obviously upregulated in the early stage. *P ≤ 0.05; NS: no significant difference.

These mistakes do not affect the results of this study. The authors apologize for these errors.

