# Author Correction: Exosomes derived from human adipose mensenchymal stem cells accelerates cutaneous wound healing via optimizing the characteristics of fibroblasts

**DOI:** 10.1038/s41598-026-36531-0

**Published:** 2026-01-21

**Authors:** Li Hu, Juan Wang, Xin Zhou, Zehuan Xiong, Jiajia Zhao, Ran Yu, Fang Huang, Handong Zhang, Lili Chen

**Affiliations:** Department of Stomatology, Union Hospital, Tongji Medical College, Huazhong University of Science and Technology, Wuhan, 430022 Hubei China

Author Correction to: *Scientific Reports* 10.1038/srep32993, published online 12 September 2016

This article contains an error in Figure 6.

As a result of an error during figure assembly, the wound closure image for the PBS group at Day 7 in Figure 6A mistakenly displays a duplication of the PBS group for Day 5.

The correct Fig. 6 and accompanying legend appear below.

**Fig. 6:**
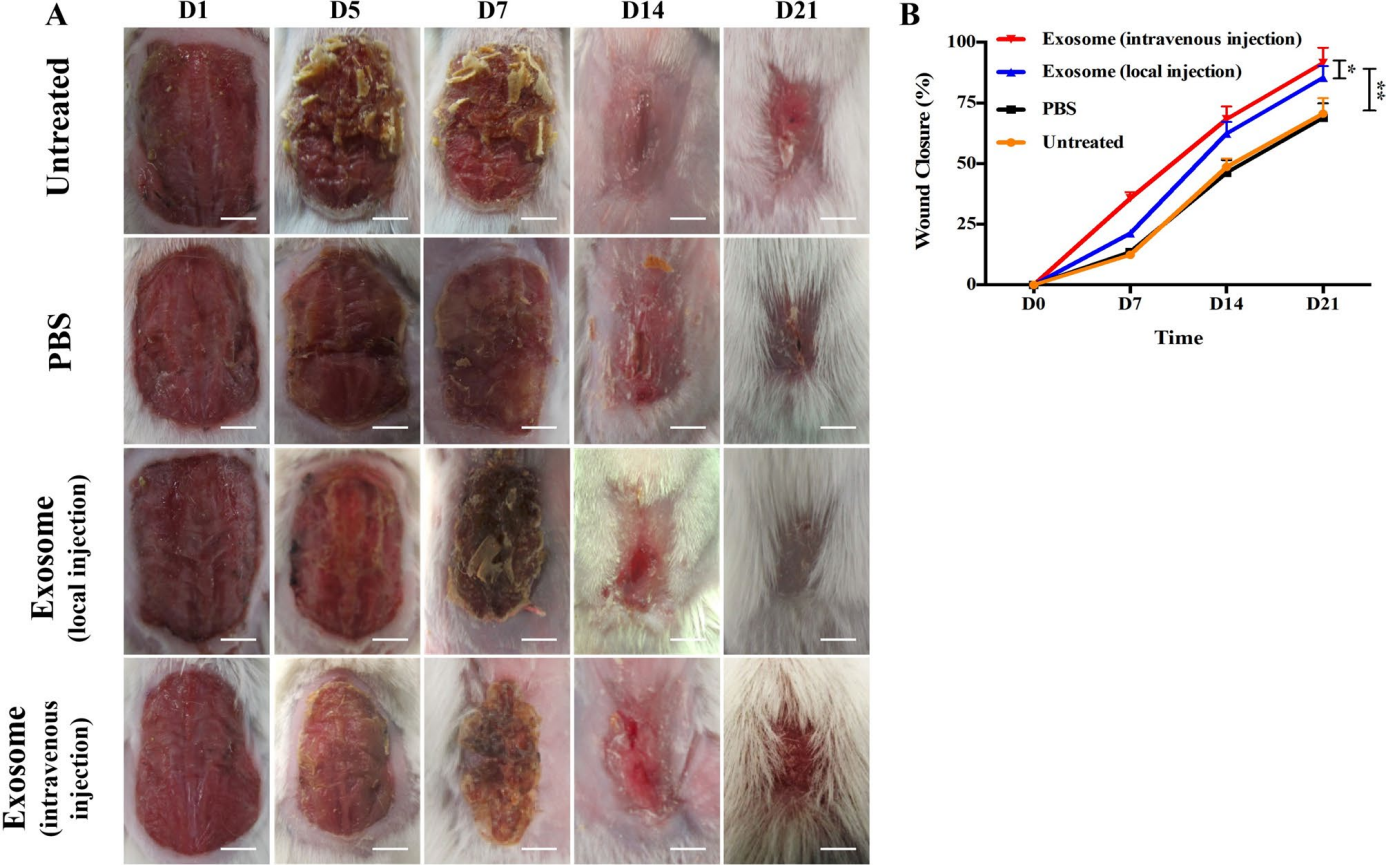
ASCs-Exos promoted wound healing in vivo. Representative wound closure imaging of animals injected locally or intravenously with exosomesor PBS after sufferring a back wound (2 × 1.5 cm) were collected at indicated time points of Day 1, 5, 7, 14, 21 (**A**). Untreated and PBS injection severed as control. Quantitative analysis of wound closure at different time points (**B**). *P < 0.05, **P < 0.01.

