# Supplementary material for: Exosomes derived from human adipose mensenchymal stem cells accelerates cutaneous wound healing via optimizing the characteristics of fibroblasts
Source: Sci Rep. 2016 Sep 12;6:32993. doi: 10.1038/srep32993 (PMC5018733; doi:10.1038/srep32993)
Supplement: Supplementary Information [file srep32993-s1.doc]

**Exosomes derived from human adipose mensenchymal stem cells accelerates cutaneous wound healing via optimizing**

**the characteristics of fibroblasts**

Li Hu^1,*^, Juan Wang^1,*^, Xin Zhou^1,*^, Zehuan Xiong^1^, Jiajia Zhao^1^, Ran Yu^1^, Fang Huang^1^, Handong Zhang^1^, Lili Chen^1^

^1^ Department of Stomatology, Union Hospital, Tongji Medical College, Huazhong University of Science and Technology, Wuhan, Hubei 430022, China

^*^ These authors contributed equally to this work.

Correspondence and requests for materials should be addressed to L.C (email: [chenlili@whuh.com](mailto:chenlili@whuh.com))

**
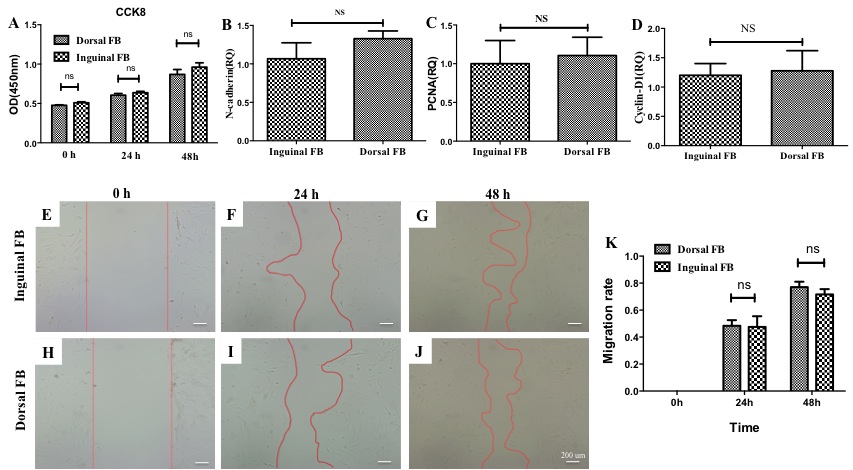
**

**Supplementary Figure 1. Comparision of proliferation and migration abilities of mice inguinal and dorsal fibroblasts in vitro**. Mice inguinal and dorsal fibroblasts proliferation were detected with a CCK-8 kit at 24 hours and 48 hours culture (**A**). The N-calcium (**B**), PCNA (**C**) and Cyclin-1 (**D**) mRNA expression were determined by RT-PCR. Mice inguinal and dorsal fibroblasts migrations were detected by Scratch closure test (**E - J**), and their migration rate were calculated (**K**).


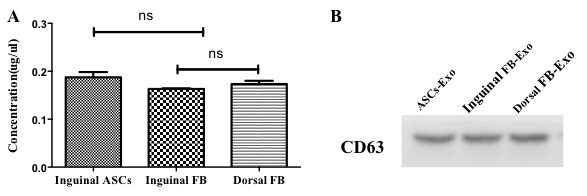


**Supplementary Figure 2. Concentrations of exosomes secreted by mice inguinal ASCs, inguinal fibroblasts and dorsal fibroblasts.** There is no significant different among exosomes concentrations of these three different cells (**A**). Similar protein level of CD63 existed in three different cells (**B**).
